# Adropin Improves Radiation-Induced Myocardial Injury via VEGFR2/PI3K/Akt Pathway

**DOI:** 10.1155/2022/8230214

**Published:** 2022-06-29

**Authors:** Bingda Li, Zhenhua Wang, Yuanqiao He, Tianpeng Chen, Yun Zhang, Xingxing Yuan, Ping Li

**Affiliations:** ^1^Department of Cardiovascular Medicine, The Second Affiliated Hospital of Nanchang University, Nanchang, Jiangxi 330006, China; ^2^Department of Laboratory Animal Science, Nanchang University, Nanchang, Jiangxi 330031, China; ^3^Department of Pharmacy, Fengcheng City People's Hospital, Yichun City 331100, China; ^4^Department of Radiation Oncology, Jiangxi Cancer Hospital of Nanchang University, Nanchang, Jiangxi 330029, China

## Abstract

Mediastinal cancer radiotherapy exposes the heart and causes myocardial injury. It is of utmost importance to identify effective prevention and treatment targets. In this study, the regulatory role of adropin (Ad) in radiation-induced myocardial injury (RIMI) was explored in mice. After C57BL/6 mice were administered E0771 cells and received radiotherapy, the effects of exogenous Ad intervention on myocardial fibrosis, apoptosis, microvessel density, oxidative stress, and protein expression levels were observed. The results showed that exogenous Ad effectively improved cardiac function, suppressed oxidative stress, inhibited myocardial fibrosis, reduced myocardial apoptosis, and promoted microangiogenesis in RIMI mice. Ad also downregulated the expression levels of transforming growth factor *β*1 (TGF-*β*1), NADPH oxidase 4 (NOX4), and cleaved caspase 3 and upregulated the expression of phosphor-endothelial nitric oxide synthase (p-eNOS). However, the above-mentioned effects of Ad were significantly reversed in Ad^−/−^ mice. Radiotherapy resulted in the downregulation of phosphor-vascular endothelial growth factor receptor (p-VEGFR2) and p-Akt in myocardial tissue, which were upregulated by Ad. However, after targeted inhibition of VEGFR2 with apatinib, the effect of Ad on improving RIMI was significantly reversed. Taken together, exogenous Ad significantly ameliorated RIMI by reducing oxidative stress, promoting microangiogenesis, and inhibiting myocardial fibrosis and apoptosis. The underlying molecular mechanism involved may be elucidated by activation of the VEGFR2/PI3K/Akt pathway.

## 1. Introduction

Radiotherapy is a currently important adjuvant treatment for malignant tumors, which can significantly prolong the survival time and quality of life of patients. However, it is also an important cause of cardiovascular dysfunction [1]. Radiotherapy mainly generates reactive oxygen species (ROS) by reacting with H_2_O to target multiple intracellular organelles (such as DNA, mitochondria, and cell membranes) to deliver ionizing radiation (IR) to destroy cancer cells [2]. When the radiotherapy target is in the mediastinum area, the heart is unavoidably exposed, thereby causing radiation-induced heart disease (RIHD), including hypotension, pericardial disease, myocardial choking, myocardial fibrosis, coronary artery disease, and valvular disease [2, 3]. The examination, monitoring, and treatment of cardiovascular diseases during cancer treatment have attracted increased attention. Avoiding exposure of the heart and reducing the radiation dose as far as possible is a common strategy for protection of the heart in radiotherapy [2]. Drugs, such as statins, have been proven to prevent RIHD [4]. The detailed mechanism of radiation-induced myocardial injury (RIMI) is not yet fully understood. Therefore, it is of utmost importance to identify effective prevention and treatment targets.

Adropin (Ad) is a highly conserved 76-amino acid peptide hormone, encoded by an energy homeostasis-related gene (Enho) that was first discovered in mice by Kumar et al. [5]. Early studies have shown that Ad is involved in the regulation of energy homeostasis and metabolism [5–8]. In addition to the pancreas, liver, brain, and kidney, Ad is expressed in the endocardium, myocardium, and epicardium [9]. Cardiac energy metabolism is closely related to the occurrence and development of many heart diseases. In previous studies, it has been shown that the serum level of Ad is an independent predictor of a variety of cardiovascular diseases, including heart failure, myocardial infarction, and atherosclerosis [10]. Increasing evidence has shown that Ad is a potential regulator of cardiovascular function and plays a protective role in the occurrence and development of cardiovascular diseases [11, 12]. Ad can regulate the flexibility of cardiac energy substrates and protect vascular endothelial cells by increasing eNOS activity, thereby indicating that stimulating Ad signaling may be beneficial to overall cardiovascular function [13]. The role of Ad in RIMI has not yet been elucidated. In this study, the role of Ad in RIMI was investigated by using exogenous Ad and Ad^−/−^ mice.

## 2. Materials and Methods

### 2.1. Animals

Adult female/male C57BL/6 mice aged 4 to 6 weeks were purchased from Shanghai Model Organisms Center, Inc. (Shanghai, CHN), allowed to access food and water *ad libitum* and maintained under a 12 h dark/light cycle at 22°C to 25°C. Experiments were performed according to the Guide for the Care and Use of Laboratory Animals published by the U.S. National Institutes of Health (NIH Publication No. 85-23, revised in 1996) and approved and monitored by the Institutional Animal Care and Use Committee (IACUC) of Nanchang Royo Biotech Co., Ltd. (RYE2020091101).

### 2.2. Ad^−/−^ Mouse Construction

**Figure 1:**
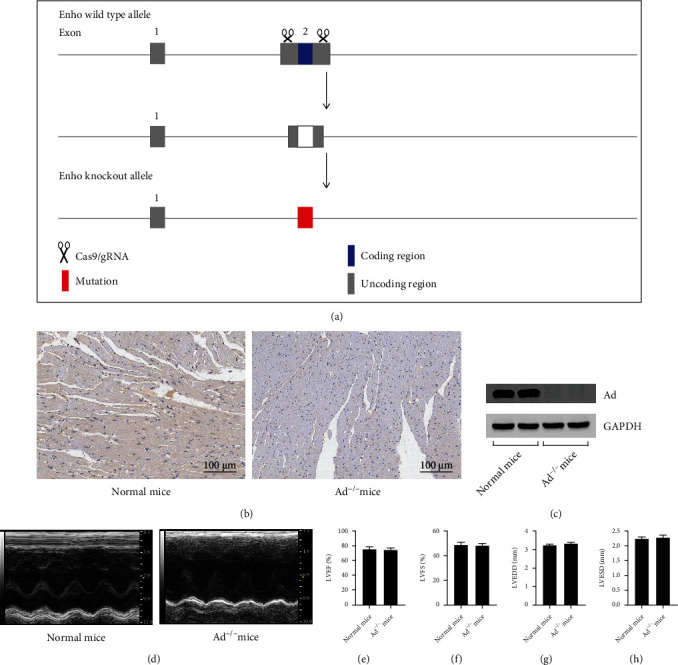
Characteristics of Ad^−/−^ mice. (a) Schematic diagram of Enho knockout obtained by CRISPR/CAS9. (b) Immunohistochemistry of Ad in myocardial tissue of normal or Ad^−/−^ mice. It can be seen that the protein expression of Ad in myocardial tissue of Ad^−/−^ mice was lost comparing with that in normal mice. (c) The western blotting was further used to detect the protein expression of Ad in myocardial tissue of normal or Ad^−/−^ mice and achieved conclusion consistent with immunohistochemistry. (d) The representative images of echocardiography of normal or Ad^−/−^ mice. (e–h) The comparison of LVEF, LVFS, LVEDD, and LVESD in normal or Ad^−/−^ mice. Values were expressed as the means ± standard error of the mean (*n* = 6 for each group).

The shift of reading frame induced by nonhomologous recombination repair introducing mutations based on CRISPR/Cas9 caused Enho gene loss of function. Briefly, Cas9 mRNA and gRNA were obtained by in vitro transcription and then microinjected into the fertilized eggs of C57BL/6J mice to obtain heterozygous mice (Ad^+/-^) (Figure 1(a)). Heterozygous mice were bred to obtain homozygous Ad knockout mice (Ad^−/−^) and identified by PCR.

### 2.3. Cell Culture and Cell-Derived Allograft (CDA) Construction

E0771 medullary breast adenocarcinoma cells were obtained from Fuheng Biology (Shanghai, CHN) and cultured in DMEM with 10% calf bovine serum at 37°C with 5% CO_2_ (*v*/*v*). The cells in logarithmic growth phase were made into 1 × 10^7^/mL cell suspension, and 100 *μ*L cell suspension was injected under the second pair of left breast pads of mice. Monitor the growth of bearing tumor.

### 2.4. Experimental Grouping and Treatment

The mice bearing tumors reaching 100-200 mm^3^ were randomly divided into the following groups. (1) The control group (Con, *n* = 12), in which the normal mice were treated with vehicle daily. (2) RIMI group (*n* = 12), in which the normal mice were intraperitoneally injected with vehicle and anesthetized by pentobarbital sodium intraperitoneally injection with dose of 80 mg/kg after 2 h. Then, the mice were exposed the bearing tumor in the supine position and 6 MV X-ray beam energy with 5 Gy dose and 300 cGy/min dose rate was used to irradiate the whole heart by a medical linear accelerator (Varian Trilogy, FL, USA) with setting source-surface distance (SSD) for 100 cm and radiation field for 1 × 1 cm. The mice were irradiated repeatedly once a day for 5 consecutive days. Radiation treatment was determined in the preliminary experiments. During and after irradiation, mice received vehicle treatment daily. (3) Ad treatment group (RIMI+Ad, *n* = 12), in which the normal mice were treated as the group of RIMI while the vehicle was replaced with 450 nmol/kg recombinant Ad^34-76^ (Qiangyao, Suzhou, JS, CHN) [14]. (4) Ad treatment in Ad^−/−^ mouse group (RIMI+Ad+Ad^−/−^, *n* = 12), in which the Ad^−/−^ mice were treated as the group of RIMI+Ad. (5) Ad^−/−^ mouse group (RIMI+Ad^−/−^, *n* = 12), in which the Ad^−/−^ mice were treated as the group of RIMI. (6) Ad and apatinib (Ap) treatment group (RIMI+Ad+Ap, *n* = 12), in which the normal mice were also orally treated with Ap (100 mg/kg/day, Hengrui Medicine Co. Ltd., Lianyungang, JS, CHN) in addition to the treatment as that in the group of RIMI+Ad. (7) Ap treatment group (RIMI+Ap, *n* = 12), in which the normal mice were treated as the group of RIMI+Ad while the Ad^34-76^ was replaced with Ap (100 mg/kg). The end point was the 6th week after the first radiation.

### 2.5. Ultrasonic Examination

Vevo 2100 imaging system (VisualSonics, Toronto, ON, Canada) was used to evaluate cardiac function of mice in each group every week during the experiment. Mice were anesthetized by inhaling isoflurane (Jiupai Pharmacy, Shijiazhuang, HB, CHN) and then fixed on a constant temperature platform in a supine position with medical tape. Use depilatory cream to remove the chest hair of mice and fully expose the heart. Apply an appropriate amount of medical ultrasound couplant, place the prepared ultrasound probe (MS400-0410) on the transverse axis of the heart, switch to the M ultrasound mode, and obtain the left ventricular short axis images during several consecutive cardiac systolic and diastolic cycles at the papillary muscle level of the largest cross section of the heart. The following indicators such as left ventricular end-diastolic diameter (LVEDD), left ventricular end-systolic diameter (LVESD), left ventricular fractional shortening (LVFS), and left ventricular ejection fraction (LVEF) were recorded accordingly.

### 2.6. Histopathological Examination

At the 6th week after the first radiation, the mice were euthanized by CO_2_ and the hearts were taken out completely. The hearts of 6 mice in each group were sliced freezingly and stained with dihydroethidium (DHE, 10 mol/L, Sigma-Aldrich; Darmstadt, GER). Ethidium fluorescence was examined by fluorescence microscopy, and the fluorescence density was calculated by Image Pro Plus 6.0 software (IPP) (Media Cybernetics, Rockville, MA, USA). The left ventricular tissue of the remaining hearts was stripped, part was fixed with formaldehyde, and part was stored in the -80°C refrigerator. After tissue was fixed overnight, it was embedded in paraffin, sectioned, and stained with hematoxylin eosin (HE), Masson's trichrome, and terminal deoxynucleotidyl transferase-mediated deoxyuridine triphosphate-biotin nick end labeling (Tunel). HE staining was used to evaluate myocardial histopathological injury. The severity of injury was evaluated with 0-4 points by pathologists blindly [15]. Masson's trichrome was used to evaluate the degree of myocardial fibrosis, and the collagen deposition area was measured by IPP. Tunel staining was used to label apoptotic cardiomyocytes, and the proportion of apoptotic cells in the total number of cells was statistically analyzed by IPP.

### 2.7. Immunohistochemistry Staining

Paraffin sections of the left ventricular myocardial tissue were incubated with bovine serum albumin, followed by incubation with anti-Ad (1 : 400, Absin, Shanghai, CHN) and anti-CD31 (1 : 400, Abcam, Cambridge, UK) overnight at 4°C. For Ad, the sections were incubated with anti-rabbit IgG (1 : 200; Proteintech, Rosemont, IL, USA) after washing for 1 h at room temperature. For CD31, the sections were then washed and incubated with CoraLite®488-conjugated anti-rabbit IgG (1 : 200; Proteintech, Rosemont, IL, USA) after washing for 1 h in dark at room temperature. After further washing, the sections were stained with DAPI for 5 min, washed, and mounted. An inverted fluorescence microscope (Leica, Wetzlar, GER) was used to capture images. IPP was used to analyze the images of immunohistochemistry staining.

### 2.8. Western Blotting

Proteins were extracted from left ventricular myocardial tissue by radioimmunoprecipitation assay (RIPA) lysis buffer and fractionated by SDS polyacrylamide gel electrophoresis. Membranes were incubated with target antibodies (Abcam) of transforming growth factor *β*1 (TGF-*β*1), NADPH oxidase 4 (NOX4), cleaved caspase 3, phosphor-eNOS (p-eNOS), eNOS, phosphor-vascular endothelial growth factor receptor (p-VEGFR2), VEGFR2, Akt, p-Akt, and GAPDH at 4°C overnight. Then, horseradish peroxidase-conjugated secondary antibodies were used to react with membranes for 2 hours at room temperature. Finally, the immune complexes were visualized by enhanced chemiluminescence and the band intensity was measured quantitatively and analyzed with the ImageJ v2.1.4.7 software (NIH, Bethesda, MD, USA).

### 2.9. Statistical Analysis

SPSS version 20.0 (IBM Corp., Armonk, NY, USA) was used to analyze experimental data presented as means ± standard error of the mean. Normally distributed data were analyzed by Student's unpaired, two-tailed *t*-test. Data not normally distributed were analyzed using the Mann–Whitney *U* test. A statistically significant difference was indicated as *P* < 0.05.

## 3. Results

### 3.1. Enho Gene Knockout Leads to Increased Susceptibility to RIMI, and Supplementation of Exogenous Ad Effectively Improves RIMI

**Figure 2:**
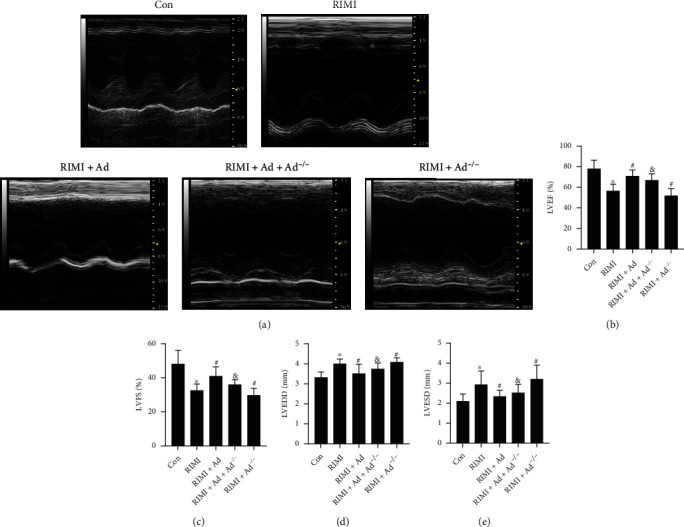
Effects of exogenous Ad and Ad knockdown on cardiac function of RIMI mice. (a) The representative images of echocardiograph in each group. (b–e) The comparison of LVEF, LVFS, LVEDD, and LVESD in each group. Values were expressed as the means ± standard error of the mean (*n* = 12 for each group). ^∗^*P* < 0.05 vs. Con group; ^#^*P* < 0.05 vs. RIMI group; ^&^*P* < 0.05 vs. RIMI+Ad group.

**Figure 3:**
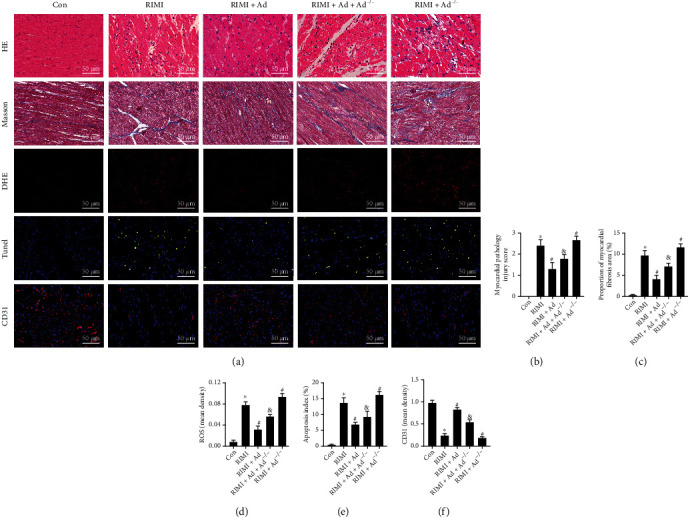
Effects of exogenous Ad and Ad knockdown on myocardial pathological injuries of RIMI mice. (a) The appearances are the representative images of the staining of HE, Masson, DHE, and Tunel and immunofluorescence of CD31 in each group. (b) Myocardial pathological injury scores in each group. (c) Proportion of myocardial fibrosis area in each group. (d) The level of ROS in myocardial tissue in each group. (e) The apoptosis indexes in each group. (f) The mean density of CD31 in myocardial tissue in each group. Values were expressed as the means ± standard error of the mean (*n* = 6 for each group). ^∗^*P* < 0.05 vs. Con group; ^#^*P* < 0.05 vs. RIMI group; ^&^*P* < 0.05 vs. RIMI+Ad group.

The effect of radiation on the cardiac function is shown in Figure 2. Radiation caused a significant decrease in LVFS and LVEF, while a significant increase in LVEDD and LVESD in mice (*P* < 0.05 vs. Con group), suggesting a significant decline in cardiac function. As shown in Figure 3, HE staining showed that radiation caused the disorder of myocardial fiber arrangement, inflammatory cell infiltration, heart valve and endocardial congestion and edema, increased fibroblasts, and significantly increased myocardial injury scores (*P* < 0.05 vs. Con group). Masson staining showed that the radiation insult resulted in excessive deposition of collagen in the myocardial tissue (*P* < 0.05 vs. Con group), suggesting the occurrence of obvious myocardial fibrosis. Radiation insult also caused excessive ROS and a large number of cardiomyocytes apoptosis (*P* < 0.05 vs. Con group). After radiation insult, the microvessel density of the myocardial tissue was significantly decreased (*P* < 0.05 vs. Con group), suggesting microvessel damage. Supplementing exogenous Ad could significantly improve the decline of cardiac function, myocardial tissue pathological injury, and myocardial interstitial fibrosis, inhibit excessive release of ROS and cardiomyocytes apoptosis, and improve microvessel density in RIMI mice caused by radiation insult (*P* < 0.05 vs. RIMI group). After knocking out Enho, it can be seen that Ad protein was lost in the mouse heart tissue, but the cardiac function of the mice was not significantly different from that of the normal mice (Figures 1(b)–1(h), *P* > 0.05 vs. Con group). However, compared with normal mice, Ad^−/−^ mice after radiation insult showed more severe myocardial injury, including cardiac function indicators, pathological injury, myocardial fibrosis, ROS, apoptosis, and microvascular damage (Figures 2 and 3, *P* < 0.05 vs. RIMI group). Ad^−/−^ mice also can significantly reverse the effects of exogenous Ad on RIMI in mice (*P* < 0.05 vs. RIMI+Ad group).

### 3.2. Effects of Exogenous Ad on Protein Expression Levels in Myocardial Tissue of RIMI Mice

**Figure 4:**
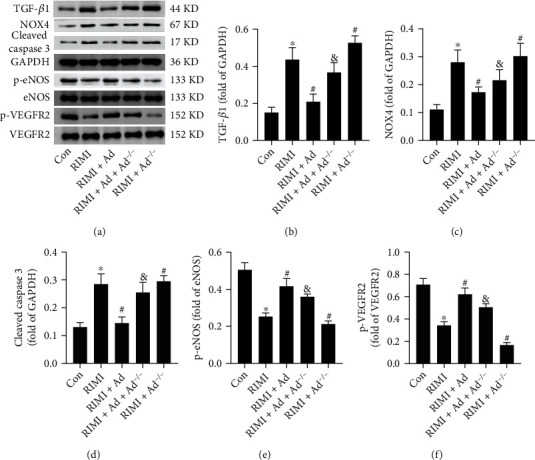
Effects of exogenous Ad and Ad knockdown on the protein expression levels of TGF-*β*1, NOX4, cleaved caspase 3, p-eNOS, and p-VEGFR2 in myocardial tissue of RIMI mice. (a) The representative bands of TGF-*β*1, NOX4, cleaved caspase 3, p-eNOS, p-VEGFR2, and GAPDH in each group. (b–f) Histogram of protein relative expression level analysis. Values were expressed as the means ± standard error of the mean (*n* = 6 for each group). ^∗^*P* < 0.05 vs. Con group; ^#^*P* < 0.05 vs. RIMI group; ^&^*P* < 0.05 vs. RIMI+Ad group.

Western blotting was used to detect the protein expression levels of TGF-*β*1, NOX4, cleaved caspase 3, p-eNOS, and p-VEGFR2 in mouse myocardial tissue. As shown in Figure 4, supplementation with exogenous Ad could significantly reverse the upregulation expression levels of TGF-*β*1, NOX4, and cleaved caspase 3 and downregulation expression levels of p-eNOS and p-VEGFR2 in mouse myocardial tissue induced by radiation insult (*P* < 0.05 vs. RIMI group). However, in the myocardial tissue of Ad knockout mice, the effects of exogenous Ad on downregulating TGF-*β*1, NOX4, and cleaved caspase 3 and upregulating p-eNOS and p-VEGFR2 were significantly attenuated (*P* < 0.05 vs. RIMI+Ad group). Compared with the RIMI group, Ad^−/−^ mice obtained higher levels of TGF-*β*1, NOX4, and cleaved caspase 3 expression and lower levels of p-eNOS and p-VEGFR2 expression after being exposed to radiation insult (*P* < 0.05 vs. RIMI group).

### 3.3. Ap Reverses the Effects of Ad on Improving RIMI

**Figure 5:**
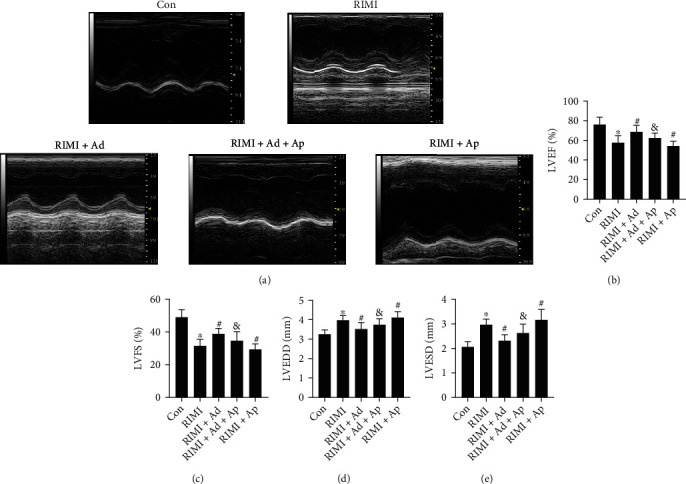
Effects of exogenous Ad and Ap on cardiac function of RIMI mice. (a) The representative images of echocardiograph in each group. (b–e) The comparison of LVEF, LVFS, LVEDD, and LVESD in each group. Values were expressed as the means ± standard error of the mean (*n* = 12 for each group). ^∗^*P* < 0.05 vs. Con group; ^#^*P* < 0.05 vs. RIMI group; ^&^*P* < 0.05 vs. RIMI+Ad group.

**Figure 6:**
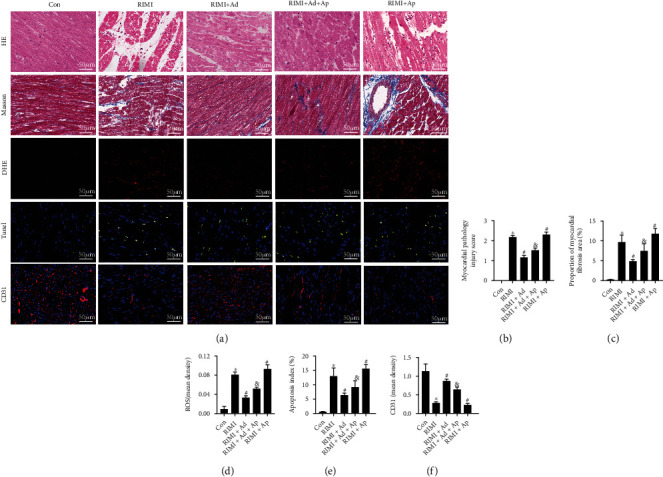
Effects of exogenous Ad and Ap on myocardial pathological injuries of RIMI mice. (a) The appearances are the representative images of the staining of HE, Masson, DHE, and Tunel and immunofluorescence of CD31 in each group. (b) Myocardial pathological injury scores in each group. (c) Proportion of myocardial fibrosis area in each group. (d) The level of ROS in myocardial tissue in each group. (e) The apoptosis indexes in each group. (f) The mean density of CD31 in myocardial tissue in each group. Values were expressed as the means ± standard error of the mean (*n* = 6 for each group). ^∗^*P* < 0.05 vs. Con group; ^#^*P* < 0.05 vs. RIMI group; ^&^*P* < 0.05 vs. RIMI+Ad group.

As shown in Figures 5 and 6, Ap could significantly reverse the effects of supplementing exogenous Ad on improving cardiac function, myocardial tissue pathological injury, myocardial interstitial fibrosis, oxidative stress, cardiomyocyte apoptosis, and myocardial microvessel density in RIMI mice (*P* < 0.05 vs. RIMI+Ad group). Ap alone could aggravate the myocardial injuries of irradiated mice (*P* < 0.05 vs. RIMI group).

### 3.4. Ap Reverses the Effects of Ad on the Protein Expression Levels in Myocardial Tissue of RIMI Mice

**Figure 7:**
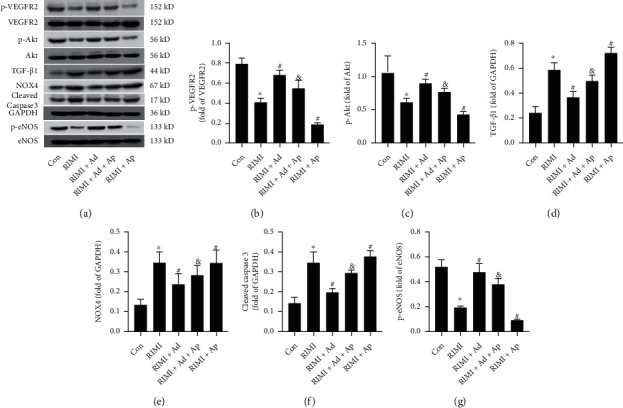
Effects of exogenous Ad and Ap on the protein expression levels of p-VEGFR2, p-Akt TGF-*β*1, NOX4, cleaved caspase 3, and p-eNOS in myocardial tissue of RIMI mice. (a) The representative bands of TGF-*β*1, NOX4, cleaved caspase 3, p-eNOS, p-VEGFR2, p-Akt, and GAPDH in each group. (b–g) Histogram of protein relative expression level analysis. Values were expressed as the means ± standard error of the mean (*n* = 6 for each group). ^∗^*P* < 0.05 vs. Con group; ^#^*P* < 0.05 vs. RIMI group; ^&^*P* < 0.05 vs. RIMI+Ad group.

As shown in Figure 7, supplementation with exogenous Ad could significantly downregulate the expression levels of TGF-*β*1, NOX4, and cleaved caspase 3 and upregulate the expression levels of p-eNOS, p-VEGFR2, and p-Akt in the myocardial tissue of mice after radiation insult (*P* < 0.05 vs. RIMI group). Ap could significantly reverse the above-mentioned effects of Ad on the protein expression levels of TGF-*β*1, NOX4, cleaved caspase 3, p-eNOS, p-VEGFR2, and p-Akt (*P* < 0.05 vs. RIMI+Ad group). In addition, compared with the RIMI group, Ap alone could further upregulate the expression levels of TGF-*β*1, NOX4, and cleaved caspase 3 and downregulate the expression levels of p-eNOS, p-VEGFR2, and p-Akt (*P* < 0.05 vs. RIMI group).

## 4. Discussion

Ad is a peptide hormone regulated by nutrients and plays a physiological role in energy homeostasis and metabolic regulation [5]. Ad has systemic effects on insulin sensitivity and glucose metabolism in the heart, liver, and skeletal muscle. The cardiovascular function of Ad has attracted attentions in recent years, which is closely related to coronary heart disease, cardiac syndrome X, heart failure, and hypertension [16]. Ad can regulate the flexibility of cardiac energy substrates and protect vascular endothelial cells by increasing eNOS activity and reducing inflammatory response [17]. Ad can also regulate the expression of fibronectin nuclear protein in vascular smooth muscle cells through phosphatidylinositol 3 kinase (PI3K), thus affecting plaque stability and vascular elasticity [17]. Moreover, Ad decreased the expression of mitochondrial acetyltransferase GCN5L1, thereby resulting in decreased acetylation of PDH and increased blood glucose utilization in DIO mice [18]. Ad treatment can improve the function and efficiency of the isolated heart and stimulate downstream activation of cardiac insulin signals through MAPK and FOXO1 signals, thereby increasing glucose uptake and utilization in lean mice [12]. Therefore, Ad is considered a potential target for cardiovascular protective therapy [13]. In this study, Ad^−/−^ mice were successfully constructed and no significant changes were observed in ultrasonic cardiac function compared with healthy (normal) mice. After radiation, Ad^−/−^ mice presented with worse cardiac function indexes compared to normal mice. These results showed that the absence of Ad under normal circumstances has no obvious effect on cardiac function, but weakens the heart's resistance to adverse factors, such as radiation. Intervention with exogenous Ad can significantly improve RIMI, indicating that exogenous supplementation of Ad is a potential treatment strategy.

Radiotherapy is a necessary method for the treatment of various tumors. Although the development of precision-directed radiation technology has reduced radiation-caused damage to healthy tissues, collateral damage caused by radiotherapy is still a significant concern. RIHD is common incidental radiotherapy-induced injury of, especially in patients with breast cancer, lung cancer or mediastinal lymphoma due to the inevitable direct exposure of the heart during radiotherapy, making them a high-risk group of RIHD [19]. Myocardial fibrosis is the main common end point of RIHD [20]. Fibrosis contributes to abnormal myocardial remodeling, resulting in reduced ejection fraction and heart failure [21]. Radiation-induced vascular injury is the root cause of myocardial fibrosis, and the imbalance of ROS and NO is the main cause of vascular injury after radiation [21]. Following radiation exposure, NADPH oxidase is upregulated, especially NOX4 in vascular endothelial and cardiomyocytes, which promotes the excessive production of ROS [22]. NO is directly scavenged by ROS and peroxynitrite is formed, which triggers nitrosylation of protein complexin residues and lipid peroxidation, ultimately resulting in impaired vasomotor responses and narrowing of blood vessels with loss of capillaries [23]. Reduced vessel density leads to ischemia, cardiomyocyte apoptosis, and fibrosis. In this study, we observed reduced cardiac function and massive release of ROS in myocardial tissue after irradiation. The pathological results showed obvious pathological myocardial injury, excessive collagen deposition, microvascular reduction, and cardiomyocyte apoptosis. Ad intervention can significantly improve the above-mentioned adverse radiation-caused insults by improving cardiac function and significantly downregulating the expression levels of TGF-*β*1, NOX4, and cleaved caspase 3 in the myocardium and increase eNOS activity; all of which could be reversed in Ad^−/−^ mice. In addition, radiation insult was markedly aggravated in Ad^−/−^ mice. These results illustrate that Ad plays a very critical role in protecting the heart from RIMI. The absence of Ad can aggravate RIMI, whereas exogenous supplementation of Ad is able to effectively ameliorate RIMI and may be related to its regulation on expression levels of TGF-*β*1, NOX4, cleaved caspase 3, and p-eNOS.

Previous studies have shown that intracellular signal transduction pathways triggered by Ad may include NB3, Notch, VEGFR2, SIRT1, cAMP, and PLC [16]. Furthermore, western blot results showed that the expression level of p-VEGFR2 in mouse myocardial tissue was significantly downregulated after irradiation, thereby suggesting that the VEGFR2 signaling pathway was inhibited. VEGFR2 is a subtype of vascular endothelial growth factor family receptors, which can stimulate endothelial cell migration, proliferation, and duct formation to regulate endothelial cell function and angiogenesis [24]. Indirect activation of VEGFR2 signaling in cardiac endothelial cells induces angiogenesis and angiocrine release of ErbB receptor ligands [25]. Physiological doses of ROS are involved in the migration and proliferation of endothelial cells mediated by VEGFR2, while excess ROS has the opposite effect [26]. Transplanting endothelial progenitor cells in the area of myocardial infarction can activate the VEGFR2-PI3K/Akt-eNOS pathway to increase microvessel density, reduce interstitial fibrosis, and improve myocardial infarction [27]. In cerebral ischemia-reperfusion injury, activation of VEGFR2 can protect brain endothelial cells through PI3K/Akt intracellular pathways, inhibit excessive ROS production, and improve angiogenesis [28]. In a rat model of renal fibrosis with unilateral ureteral obstruction (UUO), renal microvascularization was increased and renal fibrosis was delayed by activating the VEGFR2 pathway [29]. Lovren et al. showed that VEGFR2 is required for signal transduction of Ad in cardiomyocytes, thus indicating that this protein may act as a receptor for Ad [11]. In this study, expression levels of p-VEGFR2 and p-Akt protein were significantly upregulated in RIMI mice after Ad intervention, but were significantly reversed by the VEGFR2 inhibitor Ap as well as the effects of Ad on improving RIMI. The above results indicate that the effect of Ad on improving RIMI may be achieved by activating the VEGFR2/PI3K/Akt signaling pathway.

**Figure 8:**
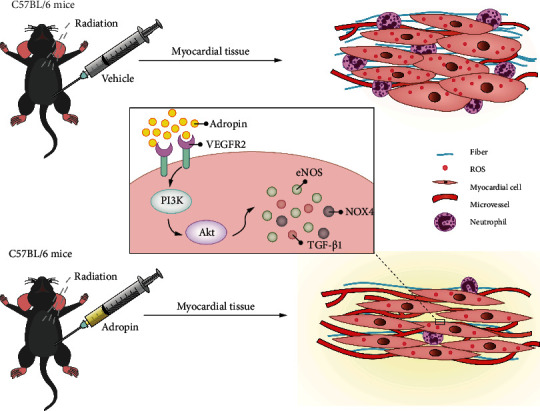
The schematic diagram of this study.

In conclusion, in this study, a RIMI mouse model was established and it was observed that exogenous Ad intervention could significantly inhibit overproduction of radiation-induced myocardial ROS, inhibit apoptosis, promote microangiogenesis, and improve myocardial fibrosis. Ad also downregulated the expression levels of TGF-*β*1, NOX4, and cleaved caspase 3 in the myocardium and increased eNOS activity. However, the above-mentioned effects can be reversed in Ad^−/−^ mice. Taken together, these results suggest that Ad plays a key regulatory role in RIMI. Furthermore, Ad could activate the VEGFR2/PI3K/Akt pathway, and the interference of Ap reversed the effect of Ad on improving RIMI. We speculate that the effect of Ad on ameliorating RIMI is achieved by acting on VEGFR2 and activating the intracellular PI3K/Akt pathway (Figure 8).

## Data Availability

The raw data supporting the conclusions of this article will be made available by the authors, without undue reservation.
